# Hands-On Times, Adherence to Recommendations and Variance in Execution among Three Different CPR Algorithms: A Prospective Randomized Single-Blind Simulator-Based Trial †

**DOI:** 10.3390/ijerph17217946

**Published:** 2020-10-29

**Authors:** Sami Rifai, Timur Sellmann, Dietmar Wetzchewald, Heidrun Schwager, Franziska Tschan, Sebastian G. Russo, Stephan Marsch

**Affiliations:** 1 Department of Orthopedics and Trauma Surgery, Bethesda Hospital, 47053 Duisburg, Germany; s.rifai@t-online.de; 2 Department of Orthopedics, 360 Clinic, 40882 Ratingen, Germany; 3 Department of Anaesthesiology and Intensive Care, Bethesda Hospital, 47053 Duisburg, Germany; t.sellmann@bethesda.de; 4 Department of Anaesthesiology, University of Witten/Herdecke, 58448 Witten, Germany; sebastian.russo@sbk-vs.de; 5 Institution for Emergency Medicine, 59755 Arnsberg, Germany; wetzchewald@aim-arnsberg.de (D.W.); h.schwager@cardio-tours.de (H.S.); 6 Department of Psychology, University of Neuchatel, 2000 Neuchâtel, Switzerland; franziska.tschan@unine.ch; 7 Department of Anaesthesiology, Villingen-Schwenningen Hospital, 78052 Villingen-Schwenningen, Germany; 8 Department of Anaesthesiology, University of Goettingen, 37075 Goettingen, Germany; 9 Department of Intensive Care, University Hospital, 4031 Basel, Switzerland

**Keywords:** cardiopulmonary resuscitation (CPR), guidelines, cardiocerebral resuscitation, adherence, simulation, randomized trial

## Abstract

Background: Alternative cardiopulmonary resuscitation (CPR) algorithms, introduced to improve outcomes after cardiac arrest, have so far not been compared in randomized trials with established CPR guidelines. Methods: 286 physician teams were confronted with simulated cardiac arrests and randomly allocated to one of three versions of a CPR algorithm: (1) current International Liaison Committee on Resuscitation (ILCOR) guidelines (“ILCOR”), (2) the cardiocerebral resuscitation (“CCR”) protocol (3 cycles of 200 uninterrupted chest compressions with no ventilation), or (3) a local interpretation of the current guidelines (“Arnsberg“, immediate insertion of a supraglottic airway and cycles of 200 uninterrupted chest compressions). The primary endpoint was percentage of hands-on time. Results: Median percentage of hands-on time was 88 (interquartile range (IQR) 6) in “ILCOR” teams, 90 (IQR 5) in “CCR” teams (*p* = 0.001 vs. “ILCOR”), and 89 (IQR 4) in “Arnsberg” teams (*p* = 0.032 vs. “ILCOR”; *p* = 0.10 vs. “CCR”). “ILCOR” teams delivered fewer chest compressions and deviated more from allocated targets than “CCR” and “Arnsberg” teams. “CCR” teams demonstrated the least within-team and between-team variance. Conclusions: Compared to current ILCOR guidelines, two alternative CPR algorithms advocating cycles of uninterrupted chest compressions resulted in very similar hands-on times, fewer deviations from targets, and less within-team and between-team variance in execution.

## 1. Introduction

Based on international consensus of collaborating partners in ILCOR (International Liaison Committee on Resuscitation), national and regional resuscitation organizations regularly publish guidelines for cardiopulmonary resuscitation (CPR) [1,2]. Despite slight improvements, the outcome of victims of cardiac arrest has remained poor [3,4,5], which prompted the development of alternative CPR algorithms for adults. One such example is the cardiocerebral resuscitation (CCR) protocol, introduced for primary out-of-hospital cardiac arrests (OHCA), i.e., arrests of cardiac origin. CCR stipulates three cycles of 200 uninterrupted chest compressions, each followed by rhythm analysis and defibrillation, if indicated. No active ventilation takes place during the first three cycles [6,7,8,9]. Compared with the pre-implementation period CCR was associated with improved neurologically intact survival of patients with OHCA in large US regions [10].

Likewise, with the goal of enabling uninterrupted chest compressions, a local interpretation of the CPR guidelines was developed in Arnsberg, Germany. The Arnsberg algorithm advocates the immediate insertion of a supraglottic airway, followed by cycles of 200 uninterrupted chest compressions, separated by rhythm analysis and defibrillation, if indicated. The rationale for choosing a supraglottic airway instead of endotracheal intubation are the shorter time for insertion and the observation that uninterrupted chest compression may follow directly after insertion [2,11]. By contrast to CCR, ventilation is applied right from the start via the supraglottic airway, so that this algorithm is applicable to both primary and secondary (e.g., asphyxia-induced) arrests. The Arnsberg algorithm has found broad regional acceptance and is currently employed by all ambulance services of a large county in central western Germany.

Previous research from clinical [12,13,14] and simulator-based [15,16,17,18] studies established that adherence to CPR algorithms is not optimal and that adherence varies between different components of the algorithm [14,15,19]. Insufficient adherence to CPR guidelines may significantly contribute to the persistent poor outcome after cardiac arrest [20,21]. Moreover, high variability in executing treatment algorithms may blur significant effects of treatment changes studied in clinical trials. Thus, the degree of adherence to algorithms is relevant for both individual patients and resuscitation science.

Investigating adherence to an algorithm and variability in its execution is inherently difficult in real cardiac arrests. This is especially true for the early phase of arrests as providing functional recording equipment or trained observers prior to the start of resuscitation efforts has significant practical and ethical constraints. By contrast, simulation allows the investigation of medical emergencies under realistic conditions in a highly standardized manner without endangering patients [16,17]. A particular advantage of simulation studies in cardiac arrest is the collection of data right from the start of the event. Since CPR performance shows a high agreement between real and simulated cases [12,13,17,22,23], the findings of simulator-based studies may serve as a useful alternative for research questions difficult to answer in real cases. So far, there has been no head-to-head comparison between established CPR guidelines and alternative CPR protocols and simulation appears to be the preferred method to close this scientific gap.

The aim of the present study was to compare the adherence to algorithms with hands-on time as primary outcome, of the current CPR guidelines, the Cardiocerebral Resuscitation protocol, and the Arnsberg algorithm, in simulated cardiac arrests. A further aim was to assess the effects of designated leadership on adherence and hands-on time.

## 2. Materials and Methods

### 2.1. Study Design and Setting

This is a prospective randomized single-blind trial (participants unaware of the goal of the trial), The trial was carried out following the rules of the Declaration of Helsinki and was approved by the Ethics Committee of Ärztekammer Westfalen-Lippe (2014-657-f-N) that waived the obligation to obtain consent. The trial is reported herein according to the extensions to the CONSORT statements of the Reporting Guidelines for Health Care Simulation Research [24].

### 2.2. Participants

The “Arbeitsgemeinschaft Intensivmedizin”, Arnsberg, Germany, organizes several educational courses each year for physicians from German-speaking countries working in intensive care and emergency medicine. Participants are mainly residents in their 2nd to 3rd year of clinical education in internal medicine, anesthesia, or surgery and, therefore, act at the time of our courses as (potential) first-responders in CPR. All participants of our courses were offered the opportunity to take part in voluntary simulator-based CPR workshops and informed that during their simulations they would work in teams, the simulations would be videotaped for scientific reasons and, based on the video-recordings, the performance of their teams would be analyzed in a strictly anonymous way. Physicians refusing to be filmed during the simulation were offered identical CPR workshops without videotaping and were not included in the present study.

### 2.3. Interventions

**Figure 1:**
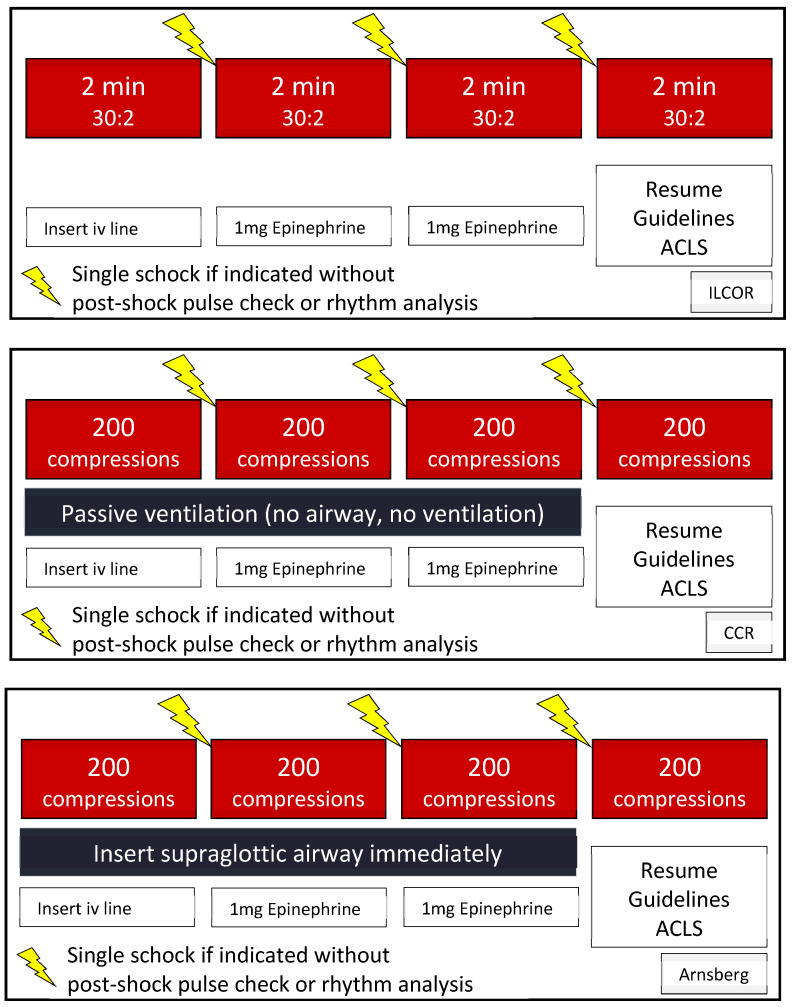
Graphical instructions handed to the participants prior to the simulated cardiac arrest. Top panel: version distributed to “ILCOR” teams. Middle panel: version distributed to the “CCR” teams. Lower panel: version distributed to the “Arnsberg” teams.

Randomizations were performed using numbered sealed opaque envelopes. Participants were randomly assigned to teams of four. Depending on the number of participants showing up at our workshops, teams of three or five were formed occasionally. Teams were randomly allocated to receive one of three versions of a graphical instruction of the resuscitation algorithm: current CPR guidelines (“ILCOR”), the Cardiocerebral Resuscitation (“CCR”) protocol (cycles of 200 uninterrupted chest compressions with no ventilation), or a local interpretation of the current guidelines (“Arnsberg”, immediate insertion of a supraglottic airway and cycles of 200 uninterrupted chest compressions). The graphical instructions were based on the Cardiocerebral Resuscitation Emergency Medical Services Protocol [9] amended by specific instructions for the different algorithms studied (“ILCOR”, “CCR”, and “Arnsberg”; Figure 1). 

Participants were asked to follow the instructions handed to them as closely as possible during the following simulated event. Except for the study-specific graphical instructions, all information given to the participants was identical. Teams were randomly allocated to designated or non-designated leadership. In designated leadership teams, the role of leader was assigned to a randomly chosen team member by the tutor immediately prior to the start of the simulation.

The mannequin Ambu Man Wireless (Ambu GmbH, Bad Nauheim, Germany) was used. All participants received a standardized introduction to the workshop and were made familiar with the mannequins used and the resuscitation equipment available. Subsequently, all team members were informed that their role during the following scenario was that of an in-hospital resuscitation team summoned to an unwitnessed cardiac arrest in their hospital’s cafeteria. The victim of the arrest (mannequin) was lying on the floor and was pulseless, apneic, and did not react to verbal commands or painful stimuli. Ventricular fibrillation could be diagnosed on the display of a manual defibrillator. The study period started with the first touch of the patient by one of the participants and ended after the third defibrillation. Trained tutors, instructed to refrain from any intervention until the end of the study period, operated the resuscitation mannequins. The further course of the scenario was at the discretion of the tutor who, after the simulated scenario, gave educational feedback to the teams.

### 2.4. Outcomes

The primary endpoint was the percentage of hands-on time, defined as time of actual chest compressions divided by the total time interval of the study period. The rationale for selecting this CPR performance measure was that hands-on time by integrating duration and interruptions of chest compressions affects clinical outcomes. [25] In “ILCOR” teams performing CPR with 30 chest compressions followed by 2 ventilations, interruptions of chest compression ≤5 sec for ventilation were counted as continuous hands-on time. A between-group difference ≥10% in hands-on time was considered to be of medical relevance. A power analysis, based on data of pilot experiments, revealed that approximately 90 teams had to be studied in each group to detect a 10% difference in the primary outcome with significance levels of 0.05 and 80% power. Accordingly, we decided to terminate the study as soon as at least 90 videotapes of sufficient quality for each group were available. For organizational reasons, the number of available videotapes of sufficient quality could be assessed only after completion of each educational course.

Secondary outcomes included adherence to the algorithm, assessed (1) on the meta-level (sequence of three blocks of chest compression separated by rhythm analysis and defibrillation); and, (2) on the level of blocks (execution according to allocation). Further secondary outcomes were number of cardiac compressions per cycle; compression rates; within-team and between team variance; and within-team changeovers for chest compressions. The effect of designated leadership was assessed as secondary outcome for all the aforementioned outcomes analyzed.

### 2.5. Analysis

Data analysis was performed using video recordings obtained during simulations. The first touch of the patient by one of the participants was defined to be the starting point for the timing of all events. All data were analyzed on an intention-to-treat basis and expressed as medians and interquartile range (IQR) unless otherwise stated. Statistical analysis was performed using SPSS (version 22). Continuous data were analyzed using analysis of variance (ANOVA), followed by Student’s *t*-test or the Mann–Whitney test with Bonferroni correction. Cohen’s d was used to calculate effect size [26]. Categorical data were analyzed using Fisher’s exact test. Coefficients of variance and Levene’s test for equality of variances were used to assess variance. A *p* < 0.05 was considered to represent statistical significance.

## 3. Results

### 3.1. Participants

**Figure 2:**
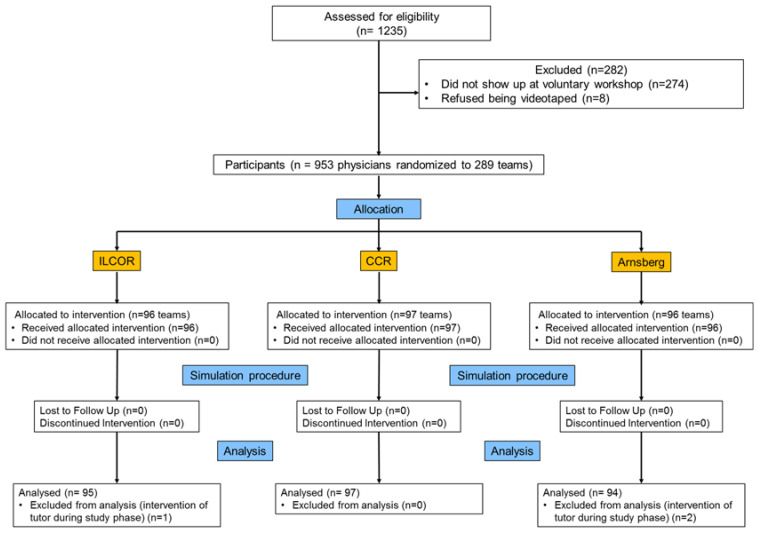
CONSORT flow chart.

Figure 2 displays the CONSORT flow chart of the trial. 940 physicians (391 male) in 286 teams (95 “ILCOR” teams, 97 “CCR” teams, and 94 “Arnsberg” teams) completed the trial.

### 3.2. Primary Outcome

**Figure 3:**
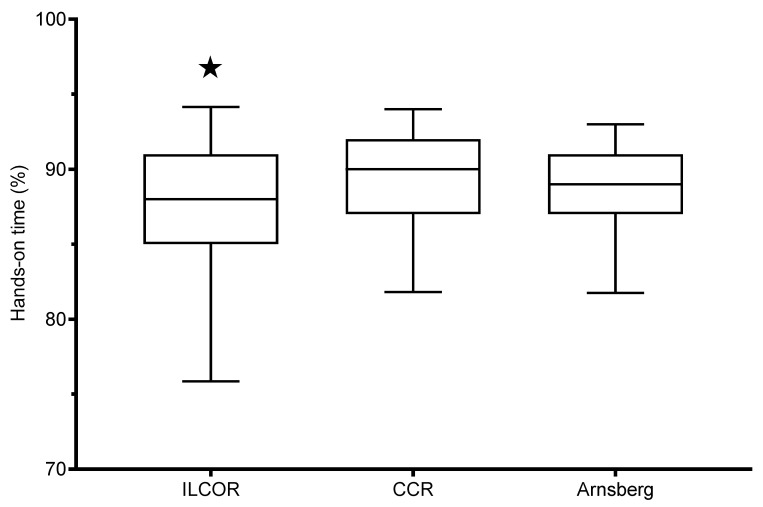
Hands-on times. Box and whiskers plot of the percentages of hands-on times, defined as time of actual chest compressions divided by the total time interval of the study period in the three study groups. Boxes indicate the median and the lower and upper quartiles while the whiskers indicate the 5th and 95th percentile. ★ = *p* < 0.05 vs. both other groups. These differences, although statistically significant, are not of medical relevance (see discussion).

Median percentage of hands-on times was 88 (IQR 6) in the “ILCOR” teams, 90 (IQR 5) in the “CCR” teams (*p* = 0.001 vs “ILCOR”, effect size 0.48), and 89 (IQR 4) in the “Arnsberg” teams (*p* = 0.032 vs. “ILCOR”, effect size 0.32; and *p* = 0.10 vs. “CCR”, effect size 0.24) (Figure 3). Designated leadership had no effect on hands-on times (*p* = 0.99).

### 3.3. Secondary Outcomes

All teams followed their instructions and performed at least four blocks of chest compressions interrupted by rhythm analysis.

**Figure 4:**
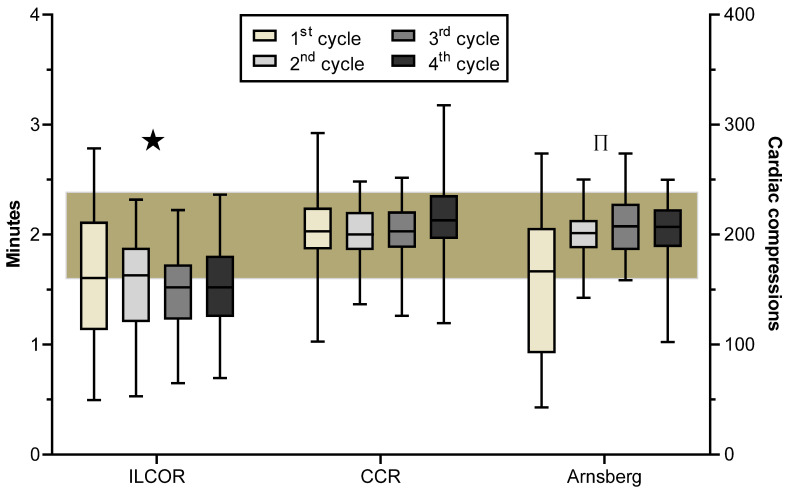
Execution of cardiopulmonary resuscitation (CPR) according to allocated target. Box and whiskers plot of the execution according to target of the allocated algorithm during the first four CPR cycles in the three study groups. Target was defined as cycle length of two minutes in the “ILCOR” teams and cycle length of 200 chest compressions in “CCR” and “Arnsberg” groups respectively. Boxes indicate the median and the lower and upper quartiles while the whiskers indicate the 5th and 95th percentile. The brownish shaded area represents the range of target ±20%. ★ = significantly (*p* < 0.001) more often outside a range of both target ± 10% and target ±20% than “CCR” and “Arnsberg” teams. ∏ = significantly (*p* < 0.001) more often outside a range of both target ±10% and target ±20% respectively than “CCR” teams.

Execution of CPR according to targets of the allocated algorithm, i.e., resuscitation cycles of 120 sec length for “ILCOR” teams and resuscitation cycles of 200 chest compressions for “CCR” and “Arnsberg” teams, respectively, is shown in Figure 4.

In the “CCR” group, 74/97 teams demonstrated complete adherence to the allocated task of not ventilating until the end of the 3rd cycle and ventilating in the 4th cycle (one team started ventilating in the 1st cycle, two in the 2nd cycle, and five in the 3rd cycle; 15 teams continued uninterrupted compressions in the 4th cycle and did not ventilate). In the “Arnsberg” group, 74/94 teams demonstrated complete adherence to the allocated task and inserted a laryngeal tube in the 1st cycle (the remaining 20 teams inserted the laryngeal tube in the 2nd cycle). Designated leadership had no significant effect on adherence to ventilation targets in the “CCR” or “Arnsberg” teams. In the “ILCOR” teams, 59 teams chose a laryngeal tube (20 teams in the 1st, 25 in the 2nd, six in the 3rd, and eight groups in the 4th cycle) and 31 teams chose endotracheal intubation (12 teams in the 1st, 11 in the 2nd, and 8 in the 4th cycle). Five teams did not perform advanced airway management. A defibrillation after each rhythm analysis was performed by 79/95 “ILCOR” teams, 84/97 “CCR” teams, and 90/94 “Arnsberg” teams (*p* = 0.014) while the remaining 33 (12%) teams did on one or more occasions (63/858 corresponding to 7.3%) not defibrillate because they did not recognize the presence of ventricular fibrillation. Designated leadership had no effect on the likelihood to defibrillate after each rhythm analysis (*p* = 0.28).

**Figure 5:**
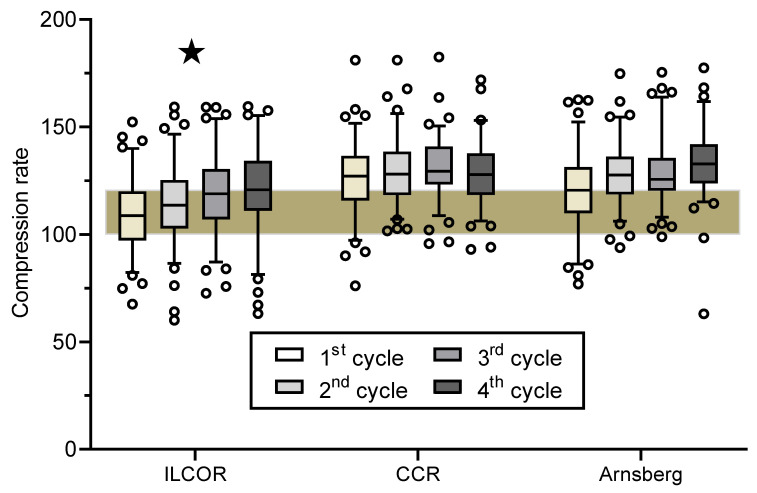
Chest compression rates. Box and whiskers plot of chest compressions rates (strokes per minute) during the first four CPR cycles in the three study groups. Boxes indicate the median and the lower and upper quartiles while the whiskers indicate the 5th and 95th percentiles. The brownish shaded area represents the range of 100–120 compressions/min recommended by the current guidelines [1,2]. ★ = *p* < 0.001 vs. both other groups.

Chest compression rates are shown in Figure 5. Chest compressions rates in “ILCOR” teams were approximately 10 compressions per minute lower than in both “CCR” and “Arnsberg” teams (*p* < 0.001) while there was no difference between “CCR” and “Arnsberg” teams (*p* = 1.0). Moreover, significantly more “ILCOR” teams had too low a number (defined as < 100/min) of delivered chest compressions in the first three cycles (cycle 1: ILCOR 28/CCR 7 Arnsberg 8, *p* < 0.001; cycle 2: 17/0/2, *p* < 0.001; cycle 3: 11/2/1, *p* = 0.003; cycle 4: 6/2/2; *p* = 0.11). “ILCOR” teams delivered in all cycles approximately 10 chest compressions less than both “CCR” and “Arnsberg” teams (*p* < 0.001). Designated leadership had no effect on delivered chest compressions (*p* = 0.65) nor on too few delivered chest compressions (*p* = 0.51).

Overall, there was a highly significant between-team difference (*p* < 0.001) and within-team difference (*p* < 0.001) for chest compression rates, length of cycles, and number of delivered chest compressions. Further analysis revealed a significant greater *within-team* variance for all three parameters in “ILCOR” teams than in “CCR” teams (*p* < 0.001) and “Arnsberg” teams (*p* = 0.02) respectively (Figure 4 and Figure 5). “Arnsberg” teams had a significantly greater within-team variance for chest compression rates (*p* = 0.001) and length of cycles (*p* = 0.012) but not number of delivered chest compressions (*p* = 0.77) than “CCR” teams.

“ILCOR” teams showed a higher between-team variance than “CCR” teams in the first three cycles (*p* < 0.001) but not in the forth cycle (*p* < 0.31), where CCR started to ventilate (Figure 4 and Figure 5). Likewise “ILCOR” teams showed higher between-team variance than “Arnsberg” teams for the second, third and fourth cycles (*p* < 0.001) but not for the first cycle (*p* = 0.16), where most of the advanced airway management of the “Arnsberg” teams took place. “CCR” teams and “Arnsberg” teams differed in between-team variance only for cycles, where airway management took place (first and fourth cycle respectively; *p* < 0.001) but not for the remaining cycles (*p* = 0.12 and 0.42). Designated leadership had no effect on within-team variance (*p* = 0.43) and between team variance (*p* = 0.58).

Current guidelines recommend a changeover or rotation of the person performing chest compressions about every two minutes corresponding approximately to the length of a compression cycle [27,28]. If followed, this would result in three or more changeovers during the studied time interval. The median observed number of changeovers was 2 (IQR 2) in “ILCOR” teams, 3 (IQR 1) in “CCR” teams (*p* = 0.012 vs. “ILCOR” teams) and 3 (IQR 1) in “Arnsberg” teams (*p* = 0.053 vs. “ILCOR” teams). A minimum of three changeovers was observed in 38/95 “ILCOR” teams, 59/97 “CCR” teams (*p* = 0.004 vs. “ILCOR”); and 52/94 “Arnsberg” teams (*p* = 0.041 vs. “ILCOR” and *p* = 0.46 vs. “CCR”).

## 4. Discussion

This prospective randomized single-blind trial assessed the adherence to and performance of three different CPR algorithms and the effects of designated leadership in simulated cardiac arrests. The cardiocerebral resuscitation algorithm resulted in higher hands-on times, less deviation from allocated targets and less variance than the currently recommended ILCOR resuscitation algorithm. An interpretation of the ILCOR algorithm advocating immediate insertion of a supraglottic airway in order to achieve cycles of 200 uninterrupted chest compressions as soon as possible also resulted in higher hands-on times, less deviation from allocated targets, and less variance than the original ILCOR algorithm. Designated leadership had a beneficial effect on between-team and within-team variance.

The observed differences in the primary outcome hands-on time of 2% between the study groups are relatively small and well below the threshold of 10% defined as representing medical relevance in our pre-trial power analysis. The resulting effect sizes of 0.32 to 0.48 indicate that approximately two thirds of the “ILCOR” teams performed below the respective averages of the “CCR” and “Arnsberg” teams [26]. While our teams achieved percentage hands-on times between 88% and 90%, previous work demonstrated no further survival benefit of compression fractions exceeding 81% [25]. Taken together, the differences in hands-on time, although statistically significant, are not of medical relevance.

As far as secondary outcomes are concerned, our results confirm substantial variance in adherence to CPR algorithms and parts thereof previously described [16,17,22,27,28]. In keeping with the results of a previous trial [28] our results confirm that temporal targets (2 min) are more difficult to follow than numerical targets (200 chest compressions). Surprisingly, only 75% of teams adhered to an unambiguous timing of airway management (no ventilation at all in the first three cycles and start of ventilation in the fourth cycle in “CCR”; immediate advanced airway management in “Arnsberg”). While immediate ventilation is undisputed as mandatory in asphyxia-induced arrests, it is up to future clinical trials to solve the ongoing controversy concerning the role and timing of advanced airway management in arrests of primary cardiac origin [29,30,31,32].

In the two CPR algorithms with a designated time of airway management (“CCR” and “Arnsberg”), within-team and between team variance for parameters related to chest compressions increased considerably in the pre-specified cycles concerned. Moreover, within-team and between team variance for parameters related to chest compressions was highest in the algorithm with constant synchronization of ventilation and chest compressions (“ILCOR”). Thus, airway management is an additional task load capable of interfering with baseline team performance. Interestingly, the disruptive effect of airway management was less pronounced when performed in the fourth cycle (“CCR”) compared to the first cycle (“Arnsberg”). We hypothesize that even a comparatively short period of team-building in de-novo forming teams has a stabilizing effect against disruptive events.

The observation that 12% of the teams did not defibrillate on one or more occasions because they did not recognize the presence of ventricular fibrillation is worrisome. Diagnostic errors in interpreting 12-lead electrocardiograms (ECG) are common, occurring in approximately 33% of cases and resulting in inappropriate management in up to 11% [33]. In a questionnaire-based study, approximately 10% of defibrillation decisions of emergency department nurses were wrong [34]. However, to the best of our knowledge, there are no real-life data on the accuracy on ECG interpretation during CPR.

Previous work demonstrated that the quality of leadership correlates with the quality of team performance during CPR and leadership skills can be successfully taught and learned [16,35,36,37,38,39,40,41]. To avoid a leadership vacuum, in many institutions the first health-care worker entering the scene at a medical emergency takes a garment that designates him/her as team leader throughout the event. The present study modelled this practice by designating one randomly chosen team member as team leader immediately prior to the encountered cardiac arrest. However, in keeping with a recent study involving medical students, the present study demonstrates no benefit of designated leadership [42]. The lack of formal CRM-training of the designated team leaders in the present study might explain differences to previous studies showing beneficial effects of leadership [16,36,37,39,40].

While (CCR) was found to improve neurologically intact survival in comparison with the pre-implementation period [10], the Arnsberg algorithm has not been tested in clinical trials so far. After the favorable results of both CCR and the Arnsberg algorithm in the present simulator-based study, comparative clinical trials against conventional guidelines would be both welcomed and justifiable. Of note, CCR was the algorithm least familiar to the study participants, so that the results for CCR might have even been better among trained rescuers.

The main goal of CPR algorithms is to maximize neurological intact survival. However, CPR algorithms should translate the current status of resuscitation science into directions for action that are easy to adhere to by potential rescuers by considering their capabilities and limitations in a stressful event. The present study demonstrates that the lower the complexity (or the higher the simplicity) of a CPR algorithm the higher the compliance with specified algorithm targets. Thus, designers of CPR guidelines should, while respecting resuscitation science, strive to achieve maximal simplicity, formulate unambiguous directions (with respect to what and when), and avoid temporal targets [28,43]. As we observed substantial between-team and within-team variance even for a presumed trivial task like counting 200 chest compressions, we advise clear directions for announcing aloud intermediate numerical targets. The present work demonstrates that prospective, randomized, blinded large-scale simulator-based trials to assess adherence to CPR algorithms are feasible. In the future, adherence to intended alterations of algorithms could thus be tested prior to definitive implementation using simulation.

Our findings relate to a scenario of a primary cardiac arrest, i.e., arrest of cardiac origin in an adult. In asphyxia-induced cardiac arrests and in arrest in the pediatric population timely and effective ventilation has the highest priority [44,45]. Strengths of our study include the large sample size and the provision of identical conditions for all teams. Limitations result from well-known limitations of simulator-based studies, which mainly include the absence of real patients and a real environment. However, this prospective large-scale comparison of different CPR algorithms would, for a variety of practical and ethical reasons, be very difficult to perform in real patients. Moreover, with regard to performance markers and the technical quality of CPR, findings in simulator-based studies show a high agreement with findings in real cases [12,13,14,17,28]

Our findings have implications for resuscitation research based on trials with a comparative or a pre-post study design. High variance and variable adherence may bias true positive effects towards the null. Moreover, it might be impossible to discern whether observed effects relate to a true effect of an intervention or merely on alterations in variance and/or adherence. A challenging task for resuscitation research remains the assessment of the impact of varying degrees of adherence to algorithms and variance in execution on patient outcomes.

## 5. Conclusions

Compared to the established ILCOR guidelines, two alternative CPR algorithms advocating cycles of uninterrupted chest compressions resulted in very similar hands-on times, but fewer deviations from algorithm targets, and less within-team and between-team variance. These simulator findings justify comparative clinical trials. Reducing the complexity of CPR algorithms may lead to a better performance and less variance. These findings may help to further optimize the translation of resuscitation science into treatment algorithms that can be easily followed by rescuers considering the stressful conditions of medical emergencies.

## References

[1] Link M.S., Berkow L.C., Kudenchuk P.J., Halperin H.R., Hess E.P., Moitra V.K., Neumar R.W., O’Neil B.J., Paxton J.H., Silvers S.M. (2015). Part 7: Adult Advanced Cardiovascular Life Support: 2015 American Heart Association Guidelines Update for Cardiopulmonary Resuscitation and Emergency Cardiovascular Care. Circulation.

[2] Soar J., Nolan J.P., Boettiger B.W., Perkins G.D., Lott C., Carli P., Pellis T., Sandroni C., Skrifvars M.B., Smith G.B. (2015). European Resuscitation Council Guidelines for Resuscitation 2015: Section 3. Adult advanced life support. Resuscitation.

[3] Goldberger Z.D., Chan P.S., Berg R.A., Kronick S.L., Cooke C.R., Lu M., Banerjee M., Hayward R.A., Krumholz H.M., Nallamothu B.K. (2012). Duration of resuscitation efforts and survival after in-hospital cardiac arrest: An observational study. Lancet.

[4] Daya M.R., Schmicker R.H., Zive D.M., Rea T.D., Nichol G., Buick J.E., Brooks S., Christenson J., MacPhee R., Craig A. (2015). Out-of-hospital cardiac arrest survival improving over time: Results from the Resuscitation Outcomes Consortium (ROC). Resuscitation.

[5] Sasson C., Rogers M.A., Dahl J., Kellermann A.L. (2010). Predictors of survival from out-of-hospital cardiac arrest: A systematic review and meta-analysis. Circ. Cardiovasc Qual. Outcomes.

[6] Ewy G.A. (2005). Cardiocerebral resuscitation: The new cardiopulmonary resuscitation. Circulation.

[7] Ewy G.A., Kern K.B. (2009). Recent advances in cardiopulmonary resuscitation: Cardiocerebral resuscitation. J. Am. Coll. Cardiol..

[8] Ewy G.A. (2012). The cardiocerebral resuscitation protocol for treatment of out-of-hospital primary cardiac arrest. Scand. J. Trauma Resusc Emerg. Med..

[9] Ewy G.A., Sanders A.B. (2013). Alternative Approach to Improving Survival of Patients With Out-of-Hospital Primary Cardiac Arrest. J. Am. Coll. Cardiol..

[10] Ewy G.A. (2017). Cardiocerebral and cardiopulmonary resuscitation—2017 update. Acute Med. Surg..

[11] Russi C.S., Miller L., Hartley M.J. (2008). A comparison of the King-LT to endotracheal intubation and Combitube in a simulated difficult airway. Prehosp. Emerg. Care.

[12] Abella B.S., Sandbo N., Vassilatos P., Alvarado J.P., O’Hearn N., Wigder H.N., Hoffman P., Tynus K., Vanden Hoek T.L., Becker L.B. (2005). Chest compression rates during cardiopulmonary resuscitation are suboptimal: A prospective study during in-hospital cardiac arrest. Circulation.

[13] Abella B.S., Alvarado J.P., Myklebust H., Edelson D.P., Barry A., O’Hearn N., Vanden Hoek T.L., Becker L.B. (2005). Quality of Cardiopulmonary Resuscitation During In-Hospital Cardiac Arrest. JAMA.

[14] Wik L., Kramer-Johansen J., Myklebust H., Sorebo H., Svensson L., Fellows B., Steen P.A. (2005). Quality of Cardiopulmonary Resuscitation During Out-of-Hospital Cardiac Arrest. JAMA.

[15] Hunziker S., Tschan F., Semmer N.K., Zobrist R., Spychiger M., Breuer M., Hunziker P.R., Marsch S.C. (2009). Hands-on time during cardiopulmonary resuscitation is affected by the process of teambuilding: A prospective randomised simulator-based trial. BMC Emerg. Med..

[16] Marsch S.C., Muller C., Marquardt K., Conrad G., Tschan F., Hunziker P.R. (2004). Human factors affect the quality of cardiopulmonary resuscitation in simulated cardiac arrests. Resuscitation.

[17] Marsch S.C., Tschan F., Semmer N., Spychiger M., Breuer M., Hunziker P.R. (2005). Performance of first responders in simulated cardiac arrests. Crit. Care Med..

[18] Tschan F., Vetterli M., Semmer N.K., Hunziker S., Marsch S.C. (2011). Activities during interruptions in cardiopulmonary resuscitation: A simulator study. Resuscitation.

[19] Marsch S., Tschan F., Semmer N.K., Zobrist R., Hunziker P., Hunziker S. (2013). ABC versus CAB for cardiopulmonary resuscitation: A prospective, randomized simulator-based trial. Swiss Med. Wkly..

[20] Crowley C.P., Salciccioli J.D., Kim E.Y. (2020). The association between ACLS guideline deviations and outcomes from in-hospital cardiac arrest. Resuscitation.

[21] Honarmand K., Mepham C., Ainsworth C., Khalid Z. (2018). Adherence to advanced cardiovascular life support (ACLS) guidelines during in-hospital cardiac arrest is associated with improved outcomes. Resuscitation.

[22] Lim S.H., Aw S.J., Cheong M.A., Chew J., Ler A.C., Yong L.P., Chan Y.H., Win M.T.M., Suppiah N. (2016). A randomised control trial to compare retention rates of two cardiopulmonary resuscitation instruction methods in the novice. Resuscitation.

[23] Wik L., Hansen T.B., Fylling F., Steen T., Vaagenes P., Auestad B.H., Steen P.A. (2003). Delaying defibrillation to give basic cardiopulmonary resuscitation to patients with out-of-hospital ventricular fibrillation: A randomized trial. JAMA.

[24] Cheng A., Kessler D., Mackinnon R., Chang T.P., Nadkarni V.M., Hunt E.A., Duval-Arnould J., Lin Y., Cook D.A., Pusic M. (2016). For the International Network for Simulation-based Pediatric Innovation, R. a. E. I. R. G. I. Reporting Guidelines for Health Care Simulation Research: Extensions to the CONSORT and STROBE Statements. Simul. Healthc..

[25] Christenson J., Andrusiek D., Everson-Stewart S., Kudenchuk P., Hostler D., Powell J., Callaway C.W., Bishop D., Vaillancourt C., Davis D. (2009). The Resuscitation Outcomes Consortium. Chest Compression Fraction Determines Survival in Patients With Out-of-Hospital Ventricular Fibrillation. Circulation.

[26] Ledesma R.D., Macbeth G., Cortada de Kohan N. (2020). Computing Effect Size Measures with ViSta—The Visual Statistics System. Tutor. Quant. Methods Psychol..

[27] Nishiyama C., Iwami T., Kawamura T., Ando M., Yonemoto N., Hiraide A., Nonogi H. (2008). Effectiveness of simplified chest compression-only CPR training for the general public: A randomized controlled trial. Resuscitation.

[28] Weichert V., Sellmann T., Wetzchewald D., Gasch B., Hunziker S., Marsch S. (2015). Two minutes CPR versus five cycles CPR prior to reanalysis of the cardiac rhythm: A prospective, randomized simulator-based trial. Resuscitation.

[29] Andersen L.W., Granfeldt A., Callaway C.W. (2017). Association between tracheal intubation during adult in-hospital cardiac arrest and survival. JAMA.

[30] Benger J.R., Kirby K., Black S., Brett S.J., Clout M., Lazaroo M.J., Nolan J.P., Reeves B.C., Robinson M., Scott L.J. (2018). Effect of a Strategy of a Supraglottic Airway Device vs Tracheal Intubation During Out-of-Hospital Cardiac Arrest on Functional Outcome: The AIRWAYS-2 Randomized Clinical Trial. JAMA.

[31] Wang H.E., Schmicker R.H., Daya M.R., Stephens S.W., Idris A.H., Carlson J.N., Colella M.R., Herren H., Hansen M., Richmond N.J. (2018). Effect of a Strategy of Initial Laryngeal Tube Insertion vs Endotracheal Intubation on 72-Hour Survival in Adults With Out-of-Hospital Cardiac Arrest: A Randomized Clinical Trial. JAMA.

[32] Granfeldt A., Avis S.R., Nicholson T.C., Holmberg M.J., Moskowitz A., Coker A., Berg K.M., Parr M.J., Donnino M.W., Soar J. (2019). Advanced airway management during adult cardiac arrest: A systematic review. Resuscitation.

[33] Breen C.J., Kelly G.P., Kernohan W.G. (2019). ECG interpretation skill acquisition: A review of learning, teaching and assessment. J. Electrocardiol..

[34] Tai C.K., Cattermole G.N., Mak P.S.K., Graham C.A., Rainer T.H. (2012). Nurse-initiated defibrillation: Are nurses confident enough?. Emerg. Med. J..

[35] Cooper S., Wakelam A. (1999). Leadership of resuscitation teams: “Lighthouse Leadership”. Resuscitation.

[36] Cooper S. (2001). Developing leaders for advanced life support: Evaluation of a training programme. Resuscitation.

[37] Fernandez C.E., Russo S.G., Cremer S., Strack M., Kaminski L., Eich C., Timmermann A., Boos M. (2011). Positive impact of crisis resource management training on no-flow time and team member verbalisations during simulated cardiopulmonary resuscitation: A randomised controlled trial. Resuscitation.

[38] Fernandez C.E., Russo S.G., Riethmuller M., Boos M. (2013). Effects of team coordination during cardiopulmonary resuscitation: A systematic review of the literature. J. Crit. Care.

[39] Fernandez C.E., Boos M., Ringer C., Eich C., Russo S.G. (2015). Effect of CRM team leader training on team performance and leadership behavior in simulated cardiac arrest scenarios: A prospective, randomized, controlled study. BMC Med. Educ..

[40] Hunziker S., Buhlmann C., Tschan F., Balestra G., Legeret C., Schumacher C., Semmer N.K., Hunziker P., Marsch S. (2010). Brief leadership instructions improve cardiopulmonary resuscitation in a high-fidelity simulation: A randomized controlled trial. Crit. Care Med..

[41] Hunziker S., Johansson A.C., Tschan F., Semmer N.K., Rock L., Howell M.D., Marsch S. (2011). Teamwork and leadership in cardiopulmonary resuscitation. J. Am. Coll. Cardiol..

[42] Hunziker S., O’Connell K.J., Ranniger C., Su L., Hochstrasser S., Becker C., Naef D., Carter E., Stockwell D., Burd R.S. (2018). Effects of designated leadership and team-size on cardiopulmonary resuscitation: The Basel-Washington SIMulation (BaWaSim) trial. J. Crit. Care.

[43] Bogenstatter Y., Tschan F., Semmer N.K., Spychiger M., Breuer M., Marsch S. (2009). How accurate is information transmitted to medical professionals joining a medical emergency?. A simulator study. Hum. Factors.

[44] Truhlar A., Deakin C.D., Soar J., Khalifa G.E., Alfonzo A., Bierens J.J., Brattebo G., Brugger H., Dunning J., Hunyadi-Anticevic S. (2015). European Resuscitation Council Guidelines for Resuscitation 2015: Section 4. Cardiac arrest in special circumstances. Resuscitation.

[45] Maconochie I.K., de Caen A.R., Aickin R., Atkins D.L., Biarent D., Guerguerian A.M., Kleinman M.E., Kloeck D.A., Meaney P.A., Nadkarni V.M. (2015). Part 6: Pediatric basic life support and pediatric advanced life support: 2015 International Consensus on Cardiopulmonary Resuscitation and Emergency Cardiovascular Care Science with Treatment Recommendations. Resuscitation.

